# Structural Properties of Quinoa Protein Isolate: Impact of Neutral to High Alkaline Extraction pH

**DOI:** 10.3390/foods12132589

**Published:** 2023-07-03

**Authors:** Shengnan Liu, Yun Xie, Bingyi Li, Siqi Li, Wenhua Yu, Aiqian Ye, Qing Guo

**Affiliations:** 1 College of Food Science and Nutritional Engineering, China Agricultural University, Beijing 100083, China; liushengnan2011@hotmail.com (S.L.); yun.xie@cau.edu.cn (Y.X.); 1h1x1f1@163.com (B.L.); 2 National Engineering Research Center for Fruit and Vegetable Processing, China Agricultural University, Beijing 100083, China; 3 Key Laboratory of Fruit and Vegetable Processing, Ministry of Agriculture and Rural Affairs, Beijing 100083, China; 4 Beijing Key Laboratory of Food Non-Thermal Processing, Beijing 100083, China; 5 Dongying Industrial Product Inspection & Metrology Verification Center, Dongying Administration for Market Regulation, Dongying 257091, China; 6 Riddet Institute, Massey University, Private Bag 11 222, Palmerston North 4442, New Zealand; s.li2@massey.ac.nz (S.L.); a.m.ye@massey.ac.nz (A.Y.); 7 Shandong Wonderful Biotech Co., Ltd., Dongying 257500, China; ywh7086@163.com

**Keywords:** plant protein, extraction conditions, structure, functionality

## Abstract

In this work, we extracted proteins from white quinoa cultivated in the northeast of Qinghai-Tibet plateau using the method of alkaline solubilization and acid precipitation, aiming to decipher how extraction pH (7–11) influenced extractability, purity and recovery rate, composition, multi-length scale structure, and gelling properties of quinoa protein isolate (QPI). The results showed that protein extractability increased from 39 to 58% with the increment of pH from 7 to 11 whereas protein purity decreased from 89 to 82%. At pH 7–11, extraction suspensions and QPI showed the similar major bands in SDS-PAGE with more minor ones (e.g., protein fractions at > 55 or 25–37 kDa) in suspensions. Extraction pH had limited effect on the secondary structure of QPI. In contrast, the higher-order structures of QPI were significantly affected, e.g., (1) emission maximum wavelength of intrinsic fluorescence increased with extraction pH; (2) surface hydrophobicity and the absolute value of zeta-potential increased with increasing extraction pH from 7 to 9, and then markedly decreased; (3) the particle size decreased to the lowest value at pH 9 and then increased to the highest value at pH 11; and (4) denaturation temperature of QPI had a large decrease with increasing extraction pH from 7/8 to 9/10. Besides, heat-set QPI gels were formed by loosely-connected protein aggregates, which were strengthened with increasing extraction pH. This study would provide fundamental data for industrial production of quinoa protein with desired quality.

## 1. Introduction

The protein plays a key role in maintaining human health as a macronutrient and structuring foods through gelation, emulsification, and foaming. Currently, there is an increasing trend in global shift towards a more plant-based food as protein sources from plants have a lower environmental impact and cost than those from most animals [1,2]. However, plant-based proteins often have lower quality than animal-based ones, triggering the debate on the efficiency and sustainability of animal vs. plant-based proteins [3,4].

Quinoa (*Chenopodium quinoa willd*) has been natively cultivated in South America for over 7000 years [5]. The high nutritional value and wide adaptability of quinoa drive a recent rapid increase of the harvested area worldwide, which is changing quinoa from a minor to major crop [6]. Furthermore, quinoa is known for its high protein content (12–17%) and superior nutritional quality (e.g., a balanced amino acid composition) [7,8,9,10,11]. Quinoa grains also contain a low content of prolamins, which makes it friendly to the population with celiac disease [12,13,14]. Therefore, quinoa proteins can be potentially used to improve nutritional and functional values of foods and beverages containing proteins.

As a seed storage protein, quinoa proteins mainly consist of 2S albumins and 11S globulins. 2S albumins account for 35% of the total protein, which are dimers of a small (3–4 kDa) and a large (8–9 kDa) subunit linked via a disulfide bridge [15]. 11S globulins (Chenopodin) account for 37% of the total protein, which are hexametric proteins of 300–450 kDa and constituted by six non–covalently bonded subunits of 50–70 kDa [i.e., an acid (20–25 kDa) and basic polypeptides (30–40 kDa) linked by disulfide bridges] [16]. 7S globulins are less abundant storage proteins in quinoa grains, which are typically trimeric proteins consisting of non-covalently bonded subunits (50–76 kDa) [13,17,18].

Quinoa protein isolate (QPI) is often extracted from quinoa grains using the alkali extraction and acid precipitation method because it can be easily used on the industrial scale. The extraction pH inevitably modifies protein structure, which in turn influences functional properties of QPI, e.g., solubility, gelling capacity, and interfacial properties [19]. Ruiz et al. found that the denaturation temperature of freeze-dried QPI extracted at pH 8–10 with 4 h stirring at room temperature and 16 h storing at 4 °C was around 97 °C; there was no endotherm peak for QPI extracted at pH 11 [20]. They also found that 10% (*w*/*w)* QPI extracted at pH 10–11 did not form self-supporting gels although extraction yield was higher at these pHs. Abugoch et al. reported that the intrinsic fluorescence emission peak wavelength was 335 and 347 nm for spray-dried QPI extracted at pH 9 and pH 11, respectively, suggesting that embedded tryptophan residues were exposed to water [21]. Mir et al. found that longer extraction times impaired functional properties of freeze-dried QPI extracted at 40 °C and varying pHs (3, 5, 7, 9, 11) [19]. Recently, research progress has been made on the effect of environmental pH on the solubility and the mechanisms of heat-induced aggregation of QPI (e.g., molecular interactions and associations of different quinoa protein fractions) [22,23,24,25]. For example, heating caused the disruption of disulfide bonds linking the acidic and basic polypeptides of the chenopodin subunits at pH 8.5 and 10.5 vs. 6.5; 11S globulins aggregated via both non-covalent interactions and disulfide bonds whereas 2S albumins were not involved in disulfide bond-induced aggregation [23,24]. However, how a wide extraction pH, especially neutral pH, influences structural properties of QPI extracted under varying conditions warrants further study, which is essential to produce quinoa protein with optimal or desired qualities.

In this study, QPI was extracted from Geli No. 1 white quinoa that has been widely cultivated in the northeast of Qinghai-Tibet Plateau of China but less studied. Extraction pH was set as 7.0, 8.0, 9.0, 10.0 and 11.0 with 90 min soaking at 25 °C. Firstly, extractability, purity, and recovery rate of quinoa proteins at different extraction pHs were investigated as well as protein composition of extraction suspensions and QPI. Secondly, CD spectra, intrinsic fluorescence, surface hydrophobicity, denaturation temperature, and particle size/zeta-potential of QPI extracted at different pHs were examined. Finally, the effect of extraction pH on solubility and heat-induced gelling properties of QPI was investigated. This study aimed to decipher how extraction pH (7–11) influenced extraction, multi-length scale structure, and gelling properties of quinoa protein, which would promote the application of plant proteins in food innovation.

## 2. Materials and Methods

### 2.1. Materials

Quinoa grains (Geli No. 1) were harvested in October 2020 and stored at 4 °C. Glycine, acrylamide, methylene bisacrylamide, NaCl, Coomassie brilliant blue, and sodium dodecyl sulfate (SDS) were purchased from Merck KGaA (Darmstadt, Germany). PageRuler Plus Pre-stained Protein Ladder (10–250 kDa) was purchased from Thermo Fisher Scientific (Waltham, MA, USA). All other reagents that were of analytical grade were purchased from Macklin Chemical (Beijing, China).

### 2.2. Quinoa Protein Extraction

Quinoa grains were washed to remove dust and saponins, and dried at 40 °C (moisture < 9.5 ± 0.5%). Dried grains were ground using a Huangdai-800g grinder (Yongkang, China) and sieved through a 60-mesh sieve. Quinoa flour was defatted using hexane at 1:4 flour:hexane (*w*/*v*) for 1 h shaking at room temperature, which was repeated four times. The defatted flour was placed in a fume hood overnight at room temperature to remove residue hexane.

The defatted flour was suspended in deionized water (10% *w*/*w*) with adjusting pH to 7.0, 8.0, 9.0, 10.0 or 11.0. These suspensions were stirred at 300 rpm and room temperature for 90 min, and then were centrifuged at 9000× *g* and 25 °C for 15 min. The supernatants were filtered through 200 mesh nylon gauze to obtain extraction suspensions.

Quinoa proteins were precipitated by adjusting the pH of extraction suspensions to 4.5 and centrifuging suspensions at 9000× *g* and 25 °C for 15 min. Remaining salts were removed or rinsed through repeating ensuing steps: the precipitates were resuspended in deionized water and centrifuged at 9000× *g* and 25 °C for 15 min. Finally, the precipitated proteins were resuspended in deionized water and neutralized using 1/6 M NaOH. QPI powder was prepared by freeze-drying QPI and grinding freeze-dried protein isolates.

### 2.3. Determination of Protein Extractability and Purity

The protein content of quinoa flours and QPI was determined by the Kjeldahl assay with a conversion factor of 5.85 [20]. The protein content of the supernatants of QPI suspensions was determined by the Bradford assay [26]. Extractability, purity, and recovery rate of quinoa proteins were calculated as follows:

$$Extractability\;\left(\%\right)=\frac{Extraction\;suspension\;volume\;\left(mL\right)\times suspension\;protein\;content\;\left(g/mL\right)}{Flour\;protein\;content\;\left(\%\right)\times flour\;weight\;\left(g\right)}\times 100$$

$$Protein\;purity\;\left(\%\right)=\frac{Isolate\;protein\;content\;\left(\%\right)\times isolate\;weight\;\left(g\right)}{Isolate\;weight\;\left(g\right)}\times 100$$

$$Recovery\;rate\;\left(\%\right)=\frac{Isolate\;protein\;content\;\left(\%\right)\times isolate\;weight\;\left(g\right)}{Extraction\;suspension\;volume\;\left(mL\right)\times suspension\;protein\;content\;\left(g/mL\right)}\times 100$$

### 2.4. Sodium Dodecyl-Sulfate Polyacrylamide Gel Electrophoresis (SDS-PAGE)

The protein composition of extraction suspensions and QPI was determined by SDS-PAGE using a Mini-PROTEAN Tetra (Bio-Rad, Hercules, CA, USA). The concentration of separating and stacking gels was 12.75 and 5%, respectively. All samples were diluted to a protein concentration of 2 mg/mL, mixed with reducing or non-reducing loading buffer with the volume ratio of 1:4, and heated in boiling water for 5 min [27]. The loading volume was 10 μL, and the voltage for stacking gels was 80 V that was changed to 120 V after samples entered separating gels. Gels were stained using Coomassie Brilliant Blue R-250 staining solution and then destained by 10% acetic acid and 45% methanol solution.

### 2.5. Structural Characterization

To obtain supernatants, 10% QPI (*w*/*v*) suspensions were centrifuged at 10,000 rpm and 25 °C for 15 min.

#### 2.5.1. Circular Dichroism (CD) Spectrum

A CD spectrometer equipped with a 0.01 cm optical path length cuvette (Chirascan, Applied Photophysics, Leatherhead, UK) was used for CD spectral analysis. The QPI supernatants were diluted to a protein concentration of 1 mg/mL using 0.01 M pH 7 phosphate buffered solution (PBS). The CD spectra of diluted supernatants were scanned in the far-UV region between 190 and 260 nm with a scanning speed of 100 nm min^−1^. The data interval was set to 1.0 nm with a bandwidth of 2.0 nm. The phosphate buffer was used as a blank. The CDNN software was used to estimate the content of α-helix, β-pleated sheet, β-turn, and unordered coils in QPI.

#### 2.5.2. Intrinsic Fluorescence Determination

Intrinsic fluorescence emission spectra were determined by a spectrophotofluorometer (Cary Eclipse, Agilent Technologies, Palo Alto, CA, USA). The QPI supernatants were diluted to a protein concentration of 1 mg/mL using 0.01 M pH 7 PBS. The excitation wavelength was 290 nm, and the slit width was set to 10 nm. The fluorescence spectra were recorded between 300 and 400 nm using a scanning speed of 600 nm min^−1^. The data pitch and slit width were set to 1.0 and 10 nm, respectively.

#### 2.5.3. Determination of Surface Hydrophobicity

1-anilinonaphthalene-8-sulfonic acid (ANS) was used as a fluorescent probe to determine the surface hydrophobicity of QPI [28]. The QPI supernatants were diluted to a protein concentration of 0.05, 0.10, 0.15, 0.20, or 0.25 mg/mL using 0.01 M pH 7 PBS. A 4 mL aliquot of diluted QPI supernatants was mixed with 20 μL of ANS solution (8 mmol L^−1^) and incubated in the dark at room temperature for 15 min. Then, fluorescence intensity was determined at an excitation wavelength of 390 nm and an emission wavelength of 470 nm on a spectrophotofluorometer (Cary Eclipse, Agilent Technologies, Palo Alto, CA, USA). The data of fluorescence intensity vs. protein concentration were fitted using linear regression. The slope of the curves indicated the surface hydrophobicity index (H_0_).

#### 2.5.4. Differential Scanning Calorimetry (DSC)

Denaturation temperatures of QPI were measured using a differential scanning calorimeter (DSC 214 Polyma, NETZSCH, Selb, Germany). 10–20 mg of the supernatant was placed in an aluminum dish which was subsequently sealed to prevent any evaporation. Water was used as the reference for this analysis. The melting procedure comprised of an equilibrium period at 25 °C for 5 min followed by a temperature ramp from 25 to 125 °C at a rate of 5 °C/min. The peak melting temperature (T_m_) was determined from DSC curves using the *Proteus* software (Version 6.1.0, NETZSCH, Selb, Germany).

#### 2.5.5. Dynamic Laser Scattering

The QPI supernatants were diluted to a protein concentration of 1 mg/mL using 0.01 M pH 7 PBS. The measurements of the particle size and zeta potential of QPI were performed on a Zetasizer Nano Series (ZEN3700, Malvern Panalytical, Malvern, UK) according to the method described in our previous study [28].

### 2.6. Determination of Protein Solubility

The pH of 1% (*w*/*v*) QPI suspensions was adjusted to 7.0 with 15 min centrifuging at 10,000 rpm and 25 °C. The Kjeldahl assay was used to measure the protein content of QPI with a conversion factor of 5.85. The protein content of QPI supernatants was measured by the Bradford assay. The protein solubility was calculated as the following equation:

$$Solubility\;\left(\%\right)=\frac{Supernanant\;protein\;content\;\left(g/mL\right)\times supernanant\;volume\;\left(mL\right)}{QPI\;protein\;content\;\left(\%\right)\times QPI\;weight\;\left(g\right)}\times 100$$

### 2.7. Dynamic Oscillatory Shear Test

QPI suspensions (pH 7) with the protein concentration of 15% (*w*/*w*) were prepared for dynamic oscillatory shear tests, which were performed using a stress-controlled rheometer (DHR-2, TA Instruments, New Castle, DE, USA) with a DIN concentric cylinder (rotor diameter: 28 mm and cup diameter: 30.36 mm). Before each test, 200 mM NaCl was added into QPI suspensions. To prevent evaporation, QPI suspensions were covered with a thin silicone oil. The heat-set gelation was induced in situ by the following procedures: (1) heating suspensions from 25 to 90 °C at a rate of 3 °C min^−1^; (2) heating at 90 °C for 30 min; (3) cooling to 25 °C at a rate of 1 °C min^−1^; and (4) cooling at 25 °C for 20 min. The storage (G′) and loss (G″) moduli were recorded as a function of time. All measurements were carried out at 0.05% strain (i.e., the linear viscoelastic region) and 1 Hz.

### 2.8. Confocal Lasering Scanning Microscopy

The microstructure of QPI gels was captured by a confocal laser scanning microscopy (LSM710, Zeiss, Jena, Germany). The gels were carefully cut into thin slices and stained by the Fast Green (0.2 wt%) for 30 min. A He–Ne laser with a wavelength of 633 nm was employed to excite the protein phase with collecting emission spectra above 650 nm.

### 2.9. Statistical Analysis

Data were analyzed using SAS, version 9.4 (SAS Institute Inc., Cary, NC, USA). One-way analyses of variance (ANOVA) with Tukey’s multiple range tests were run to test whether marked variations existed for the data of extractability, purity, recovery rate, intrinsic fluorescence, particle size, zeta-potential, surface hydrophobicity, denaturation temperature, solubility and shear moduli, at *p* < 0.05.

## 3. Results and Discussion

### 3.1. Effect of pH on Quinoa Protein Extraction

**Figure 1:**
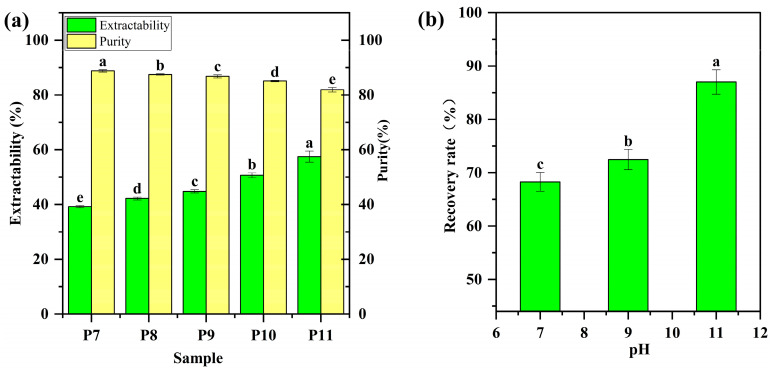
(**a**) Protein extractability and purity of quinoa protein isolate (QPI). P7, P8, P9, P10 and P11 represent QPIs extracted at pH 7, 8, 9, 10 and 11, respectively. (**b**) Recovery rate of quinoa proteins from extraction suspensions at pH 7, 8, 9, 10 and 11. Bars with different letters are significantly different at *p* < 0.05.

QPI was extracted from the defatted quinoa flour (<1% fat) at room temperature. Protein extractability increased from 39 to 58% with the increment of pH from 7 to 11 (Figure 1a), which was higher than that of sweet quinoa with longer soaking time [20]. The protein recovery rate from extraction suspensions was 68, 72, and 87% at pH 7, 9 and 11, respectively (Figure 1b), demonstrating the loss of a relatively high portion of quinoa proteins during extraction, which was one limitation of the alkali solubilization and acid precipitation method for plant protein extraction [29]. On the other hand, protein purity decreased from 89 to 82% with increasing pH from 7 to 11 (Figure 1a), which was consistent with the previous studies on protein purity of QPI extracted from other varieties of quinoa [18,20]. A high alkaline pH results in deprotonation of the amine groups, ionization of the carboxyl groups, and disruption of disulfide bonds [30,31], which could disperse and/or dissolve more protein components into extraction suspensions and subsequently increase the extractability and recovery rate of quinoa proteins. However, a high alkaline pH could simultaneously disperse and/or dissolve more non-protein components (e.g., fiber and starch) into extraction suspensions, leading to a lower protein purity [23]. Although QPI had the similar protein purity to soybean protein isolate (SPI) and pea protein isolate (PPI) that were extracted using the similar method [31,32], there was a large variation in protein extractability among these plant proteins. This suggests that extraction efficiency largely depended on the structural properties of plant proteins themselves.

**Figure 2:**
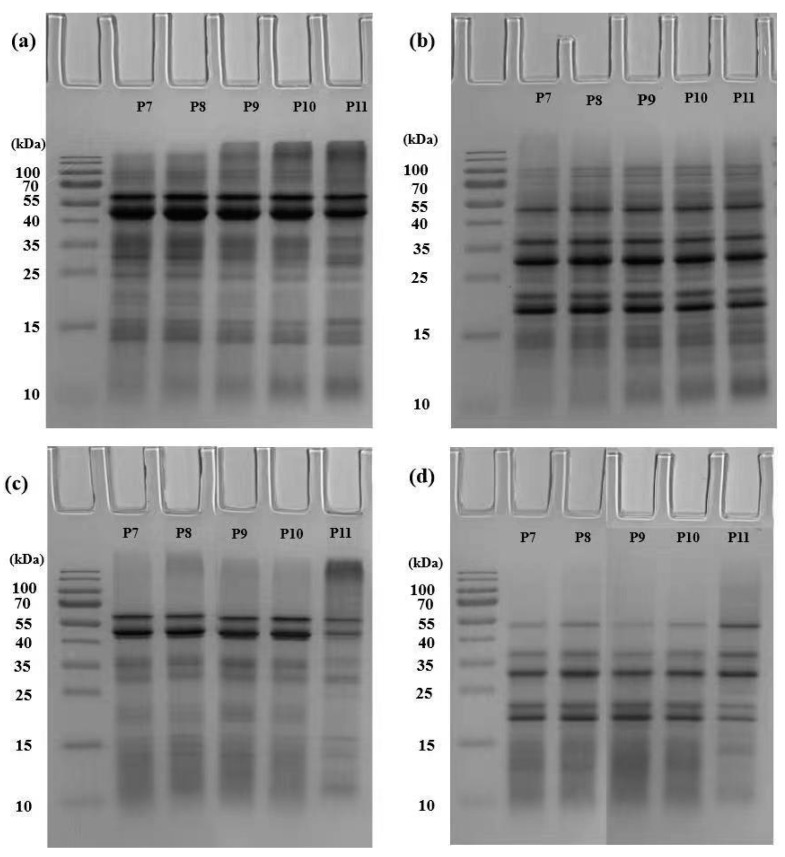
Sodium dodecyl-sulfate polyacrylamide gel electrophoresis (SDS-PAGE) of quinoa protein: (**a**) non-reducing electrophoresis of extraction suspensions; (**b**) reducing electrophoresis of extraction suspensions; (**c**) non-reducing electrophoresis of quinoa protein isolate (QPI) supernatants; (**d**) reducing electrophoresis of QPI supernatants. P7, P8, P9, P10 and P11 represent QPI extracted at pH 7, 8, 9, 10 and 11, respectively.

SDS-PAGE profiles demonstrated the composition of QPI extracted at varying pHs (Figure 2). At pH 7 to 10, extraction suspensions and QPI had the similar SDS-PAGE patterns in both reducing and non-reducing conditions. In the non-reducing condition, major bands of extraction suspensions and QPI were observed at 50–60 kDa corresponding to the subunit of 11S globulins. In the reducing condition, quinoa proteins gave doublets at 30–36 kDa and 16–19 kDa which corresponded to acidic and basic polypeptides of 11S globulin subunits, respectively. A band with molecular weight of 55 kDa was observed, which could be subunits of 7S globulins or unreduced 11S globulin subunits. The similar band was also observed in coconut and amaranth protein isolates [13,16,18,33,34]. 2S albumins were the second most abundant storage protein in quinoa grains [15], corresponding to the bands at <15 kDa in SDS–PAGE.

In the non-reducing or reducing condition, the bands had no differences between the suspensions extracted at pH 11 and other pHs. In contrast, protein aggregates of >100 kDa were observed in QPI extracted at pH 11 in the non-reducing condition. With adding DTT, these large aggregates were reduced into protein fractions at 55, 30–36, and 16–19 kDa, indicating that disulfide linkages participated in the formation of aggregates in QPI. This also suggests that non-protein components could inhibit aggregation of quinoa proteins in extraction suspensions at pH 11.

Besides, extraction suspensions and QPI had the same major bands with more minor bands (e.g., protein fractions at 25–37 or >55 kDa) in suspensions. The compositional differences between QPI and extraction suspensions demonstrate that the acid precipitation caused protein loss, which was consistent with the results of the protein recovery rate.

### 3.2. Structural Characteristics of QPI Extracted at Different pHs

**Figure 3:**
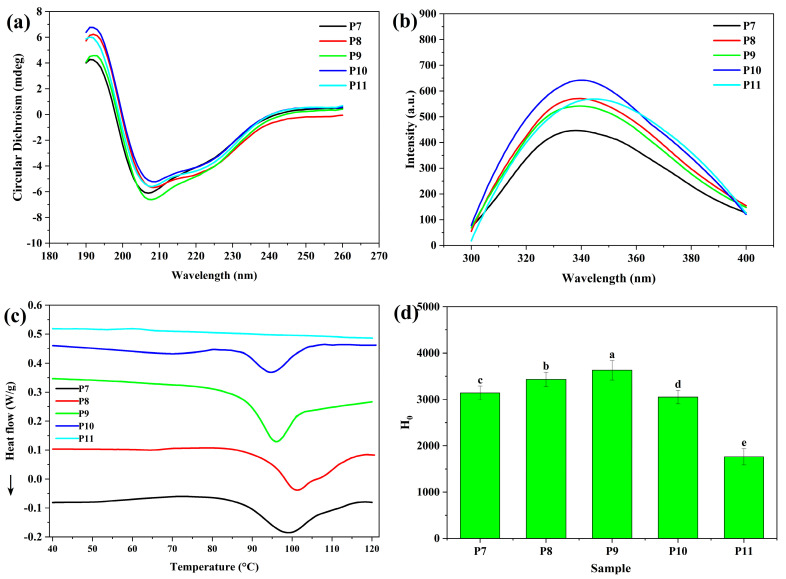
(**a**) CD spectra, (**b**) intrinsic fluorescence spectra, (**c**) DSC thermograms, and (**d**) surface hydrophobicity (H_0_) of quinoa protein isolate (QPI). P7, P8, P9, P10 and P11 represent QPI extracted at pH 7, 8, 9, 10 and 11, respectively. Bars with different letters are significantly different at *p* < 0.05.

The hierarchy structure of QPI is the key factor influencing functional properties of QPI. The effect of extraction pH on the secondary structure of QPI was evaluated using CD spectroscopy (Figure 3a). CD spectra only had slight differences between different QPI samples, indicating the secondary structure of QPI was almost retained during protein extraction regardless of pH, which was in agreement with the previous studies on different plant proteins [23,32].

The changes of fluorescence emission spectra of tryptophan residues in QPI with extraction pH are shown in Figure 3b. All fluorescence spectra of QPI showed a broad peak. The emission maximum wavelength of water-exposed tryptophan residues in proteins ranges from 305 to 350 nm, which depends on the degree of solvent exposure of the chromophore [35,36]. The emission maximum wavelength of QPI extracted at pH 7 was 338 nm (Table 1). The increase of extraction pH caused a marked red shift of the wavelength (Table 1), indicating that more embedded tryptophan residues in QPI were exposed to water with the modification of the intramolecular structure of QPI [21,35]. Compared to QPI extracted at pH 7, QPI extracted at higher pHs had greater intrinsic fluorescence intensity, suggesting that new structures could be formed due to the denaturation of QPI molecules [37]. 

Thermal properties of QPI extracted at different pHs were analyzed by DSC (Figure 3c). QPI extracted at pH 7.0, 8.0, 9.0 and 10.0 showed a single endotherm peak with denaturation temperatures at 99.3, 101.1, 95.1, and 92.4 °C, respectively; this was in line with denaturation temperatures of QPI reported in the previous studies [20,21,24]. Storage proteins from other plant seeds also have a high denaturation temperature. For instance, the denaturation temperature of globulins in chia, oat and amaranth seeds, and soybean glycinin is 105, 112, 94 and 92 °C, respectively [38,39,40,41]. Denaturation temperatures of QPI had a slight increase with increasing pH from 7 to 8, and then significantly decreased, suggesting the native structure of QPI was unfolded gradually, which was supported by the results of intrinsic fluorescence and surface hydrophobicity. QPI extracted at pH 11 had no endotherm peak, indicating that the native structure of QPI was severely disrupted during protein extraction [20,21].

The changes of surface hydrophobicity of QPI with extraction pH are shown in Figure 3d. With increasing pH from 7 to 9, surface hydrophobicity increased from 3100 to 3500, implying some aromatic amino acid residues were exposed to water, which was in agreement with the results of intrinsic fluorescence. However, with further increase of extraction pH, surface hydrophobicity sharply decreased probably because of the aggregation of QPI. The severe exposure of buried aromatic amino acid residues could induce QPI aggregation via hydrophobic interactions [42], which in turn buried aromatic amino acid residues and lowered surface hydrophobicity.

**Figure 4:**
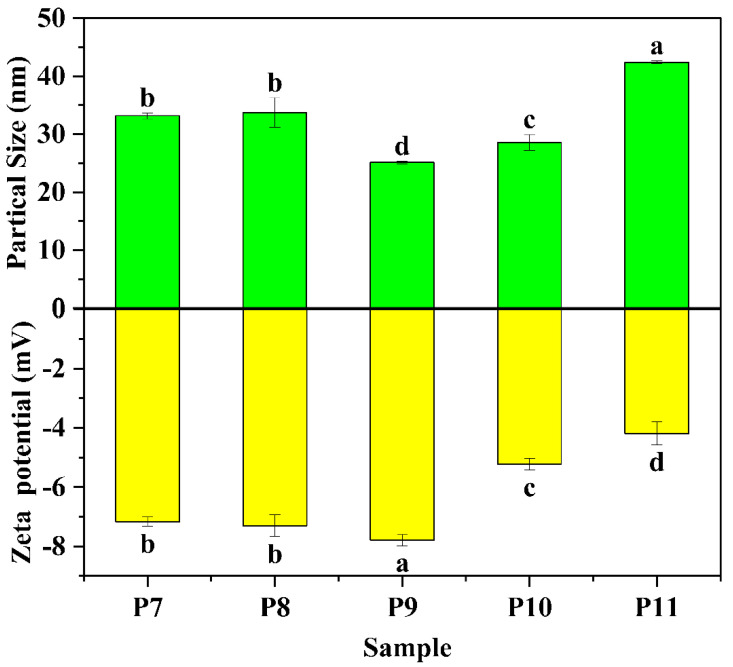
Particle size and zeta potential of quinoa protein isolate (QPI). P7, P8, P9, P10 and P11 represent QPI extracted at pH 7, 8, 9, 10 and 11, respectively. Bars with different letters are significantly different at *p* < 0.05.

The particle size and zeta-potential are important colloidal aspects of QPI, which are illustrated in Figure 4. The particle size and zeta-potential of QPI extracted at pH 7–8 had no significant differences. With increasing pH to 9, the particle size decreased probably due to the increase of surface charge, suggesting structural changes of QPI occurred (e.g., disruption of protein aggregates). When extraction pH increased to 11, the particle size and surface charge sharply increased and decreased, respectively. This demonstrated QPI molecules aggregated during extraction process because of severe protein denaturation.

Overall, the structure of QPI had limited changes at extraction pH 7–9. With increasing pH to 10, QPI had significant structural changes but still retained its main structure. At pH 11, QPI lost its native structure.

### 3.3. Solubility and Gelling Properties of QPI Extracted at Different pHs

**Figure 5:**
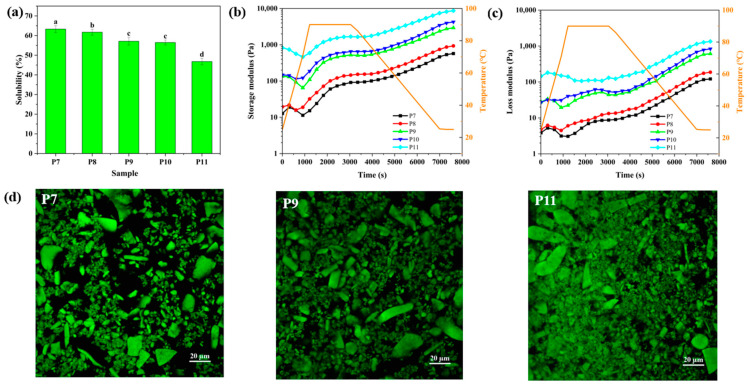
(**a**) Solubility of quinoa protein isolate (QPI), (**b**) storage and (**c**) loss moduli of QPI suspensions as a function of heating time and (**d**) microstructure of heat-set QPI gels. P7, P8, P9, P10 and P11 represent QPI extracted at pH 7, 8, 9, 10 and 11, respectively. Bars with different letters are significantly different at *p* < 0.05.

QPI solubility was determined at pH 7 (Figure 5a), which is highly related to structural properties of QPI. The solubility of QPI significantly decreased from 63 to 47% with increasing pH from 7 to 11, which was in agreement with the range of QPI solubility (35–70%) reported in the previous studies [20,21,24]. QPI extracted at pH 7 had the highest solubility probably because protein extraction was carried out at the same pH for solubility determination. As discussed in Section 3.1 and Section 3.2, denaturation of QPI molecules became increasingly severe with increasing extraction pH, subsequently resulting in protein aggregation at pH 10–11 (Figure 3 and Figure 4), which could be a key factor leading to the decrease of protein solubility.

The heat-set QPI gelation (i.e., the evolution of linear viscoelasticity as a function of heating time) was monitored by a dynamic oscillatory shear test (Figure 5b,c), which reflects the transition of structural properties of individual quinoa protein molecules to the collective macroscale properties of a network of protein molecules. Our preliminary results showed that QPI only formed very weak gels at neutral pH, which was consistent with the previous studies [20,43]. Thus, we monitored the gelation process of QPI with adding 200 mM NaCl. At the beginning of heating, QPI showed the solid-like properties with G′ > G″, which was attributed to the high protein concentration of QPI suspensions; both G′ and G″ decreased significantly probably because of the breakdown of the initial structure of QPI suspensions. With further heating, G′ and G″ increased gradually due to the heat-induced denaturation and aggregation of QPI. The final G′ of QPI gel increased from 576 to 8719 Pa with increasing pH from 7 to 11, which was ascribed to structural differences of QPI extracted at different pHs.

The microstructure of the heat-induced QPI gels is presented in Figure 5d. All gels were formed by large and irregular protein aggregates (several tens of microns) [20,23]. QPI extracted at pH 11 could form a denser gel network, which explained a higher G′ of the gel prepared by QPI extracted at pH 11. Yang et al. studied the structure of heat-set QPI gels using X-ray and neutron scattering techniques [43]. They found that heat treatment promoted aggregation of quinoa protein on the micron scale to form a fractal-like network structure at 0–200 mM NaCl concentration, which was confirmed by the result of this study. However, the G′ of QPI gels was much lower than that of fractal-like heat-set whey protein gels containing the same protein and NaCl concentrations [40,41]. This implies that thermal treatment translates structural differences between quinoa and whey proteins (e.g., lower solubility and zeta-potential, and larger molecular weight of QPI molecules) into the gel network.

## 4. Conclusions

The pH greatly influenced extraction of QPI from quinoa grains using the method of alkaline solubilization and acid precipitation, i.e., protein extractability/recovery rate and purity increased and decreased markedly with increasing extraction pH, respectively. The structure of QPI gradually changed with increasing extraction pH although there were slight changes at pH 7–9. At pH 10, the structural properties of QPI including intrinsic fluorescence, surface hydrophobicity, denaturation temperature, particle size, and zeta-potential began to change significantly. At pH 11, QPI molecules severely denatured and lost native structure. These structural changes were the key factor leading to the decrease of the solubility. However, the gel strength with respect to linear viscoelasticity increased with extraction pH, indicating that QPI translated pH-induced structural changes of QPI molecules into the gel network. By comparing these data, it is concluded that the balance between extraction conditions and protein structure/functionality should be considered in QPI extraction, which could depend on specific requirements for quinoa protein qualities. 

## Figures and Tables

**Table 1:** Intrinsic fluorescence intensity and maximum emission wavelength (λmax) of quinoa protein isolates (QPI).

Sample	P7	P8	P9	P10	P11
fluorescence intensity	446.8 ± 2.43 ^d^	570.9 ± 2.09 ^b^	541.5 ± 1.68 ^c^	642.1 ± 2.96 ^a^	569.0 ± 1.33 ^b^
λmax	338.0 ± 0.09 ^d^	339.1 ± 0.06 ^c^	340.0 ± 0.11 ^b^	340.0 ± 0.07 ^b^	345.1 ± 0.13 ^a^

P7, P8, P9, P10 and P11 represent QPI extraction at pH 7, 8, 9, 10 and 11, respectively. Different superscript letters within the same row indicate significant difference at *p* < 0.05.

## Data Availability

The data used to support the findings of this study can be made available by the corresponding author upon request.
